# Clinical, laboratory, and molecular epidemiology of *Orientia tsutsugamushi* infection from Southwestern India

**DOI:** 10.1371/journal.pone.0289126

**Published:** 2023-07-25

**Authors:** Kiran Chunduru, Manoj A. R., Subhadra Poornima, Manjunatha Hande H., Mridula M, George M. Varghese, Ramakrishna Devaki, Kavitha Saravu

**Affiliations:** 1 Department of Infectious Diseases, Kasturba Medical College, Manipal, Manipal Academy of Higher Education, Manipal, Karnataka, India; 2 Manipal Center for Infectious Diseases, Prasanna School of Public Health, Manipal Academy of Higher Education, Manipal, Karnataka, India; 3 Department of Genetics and Molecular Medicine, Kamineni Life Sciences, Hyderabad, Telangana, India; 4 Department of Medicine, Kasturba Medical College, Manipal, Manipal Academy of Higher Education, Manipal, Karnataka, India; 5 Department of Microbiology, Kasturba Medical College, Manipal, Manipal Academy of Higher Education, Manipal, Karnataka, India; 6 Department of Infectious Diseases, Christian Medical College, Vellore, Tamil Nadu, India; 7 Department of Biochemistry, Kamineni Academy of Medical Sciences and Research Centre, LB Nagar, Hyderabad, Telangana, India; Pondicherry Institute of Medical Sciences, INDIA

## Abstract

Scrub typhus is a vector borne disease which in a proportion of patients causes multiorgan involvement and death if untreated. Infecting genotype and virulence factors play a role in severity of infection and outcome. The current prospective cohort study was undertaken to elucidate the severity of illness in scrub typhus patients and to identify the circulating genotypes in Karnataka, India. A total of 214 patients of either gender from 9 districts of Karnataka and one patient each from Andhra Pradesh and Kerala, India were enrolled in the study. With a predefined severity criterion, 132 patients were segregated to the severe group. Multi organ involvement was seen in 59 (44.69%) patients. Phylogenetic analysis revealed JG-v like (48.97%), Karp-like (26.53%), JG-like (22.44%), and Kato-like (2.04%) strains in Karnataka. Patients infected with *Orientia tsutsugamushi* Karp-like strains had respiratory involvement (69.2%), cardiovascular involvement (46.2%) and thrombocytopenia (23.1%) and required higher hospital resource utilization.

## Introduction

Scrub typhus is caused by an intracellular organism *Orientia tsutsugamushi*. The infection is transmitted by the bite of the larval stage *Leptotrombidium* chigger mite. At the site of mite bite, a pathognomonic eschar appears [1, 2]. The organism was discovered in endothelial cells of the brain, heart, lung, liver, kidney, pancreas, and cardiac muscle cells, as well as macrophages of the spleen and liver, in paraffin-embedded autopsy tissue samples [3]. Focal or disseminated vasculitis is the pathologic characteristic of scrub typhus, which can cause single or multi organ dysfunction [4]. Acute respiratory dysfunction syndrome (ARDS), renal failure, hepatitis, myocarditis, and meningoencephalitis are a few complications that can occur in a subset of patients [2, 5]. The variability in clinical presentation of patients, often make the diagnosis difficult [5]. Initially, it was presumed that scrub typhus was restricted to the tsutsugamushi triangle. However, molecular evidence of *Orientia* species outside the tsutsugamushi triangle were reported from Africa, Europe, South America, and Middle East. While the serological evidence of scrub typhus in humans was reported from Africa, Middle East, and South America [6].

*Orientia tsutsugamushi* 56-kDa type-specific-antigen (TSA) is an outer membrane protein of great importance. It has a role in binding to fibronectin and the invasion of host cell [7]. The chimeric 56-kDa TSA is also used for detecting anti-scrub typhus IgM and IgG by both enzyme-linked immunosorbent assay (ELISA) and Immunochromatography test (ICT) [8, 9]. The protein is coded by a gene of ~1550 bases and the protein consist of 516–541 amino acid residues. The 56-kDa TSA gene has four variable domains (VD) designated VDI-VDIV with VDI and VDII being the most variable [10]. These VD’s antigenic variation is taken advantage of, for genotyping. Based on sequencing the DNA of 56-kDa TSA gene, nine clusters have been defined, namely, Karp-related, Saitama, Kuroki, TA763, Gilliam, Kawasaki, JG, and Kato [10]. There are a few studies in India, which have previously reported circulating genotypes. Interestingly, there are differences in circulating genotypes of *Orientia tsutsugamushi* in India [7, 11–13]. The project was undertaken, to study the severity of illness, and organ involvement, and identify the circulating genotypes of *Orientia tsutsugamushi* in Karnataka.

## Methods

### Study design and participants

A prospective cohort study was performed during August 2019 through March 2022 on adult patients admitted to Kasturba Medical College and Hospital, Manipal, Karnataka, India with suspected scrub typhus. The patients included in the study had their samples positive for either Scrub typhus IgM ELISA or IgM RDT. Detection of scrub typhus IgM was performed using Scrub typhus Direct^TM^ IgM ELISA (InBios International, Inc., Seattle, WA, USA) kit according to the manufacturer’s instructions. An IgM ELISA OD value >1.0 was used as a cut off in the patients included in this study [14]. All the patients underwent a thorough clinical assessment. To rule out other common infections seen in this geographical region, treating physicians have requested for quantitative buffy coat for malaria parasites, Weil Felix test, Widal test, Leptospira IgM, Dengue NS1 and IgM, and blood cultures. All the patients were followed up until discharge. Patients who requested discharge against medical advice in critical condition were telephonically followed up.

### Sample size calculation

Assuming the severity of scrub typhus as 30% and odds ratio of maculopapular rash OR = 1.7 in severe group and 0.9 in non-severe group and using classification of logistic regression model and P = 0.3 the required sample size was 326. By applying sample size determination using the finite population correction factor the minimum sample size required was 167.

### Laboratory procedures

The study participants clinical data and reports of laboratory investigations were recorded in a predefined case proforma. From each patient 4 ml of blood was drawn into an EDTA tube and 2 ml of blood was drawn into a plain tube. The patients and their samples were given a unique study identifiers to blind them to their identities. Blood samples collected in EDTA tubes were separated into plasma and buffy coat, while the serum was separated from the plain tube. The separated samples were transferred to cryovials and were stored at -70°C until further testing.

#### Nucleic acid amplification tests and sequencing

DNA was extracted from 300 μl of the buffy coat using an in-house DNA extraction kit which is based on salting-out method. The DNA was subjected to *Orientia tsutsugamushi* real-time PCR targeting the 112bp segment of the 56-kDa TSA gene. Simultaneously, a nested polymerase chain reaction (N-PCR) targeting the 483bp segment of the 56-kDa TSA gene was amplified. The primer pairs 34: 5’-AGGGATCCCTGCTGCTGTGCTTGCTGCG-3’ and 55: 5’-TCAAGCTTATTGCTAGTGCAATGTCTGC-3’ were used for the first round of reaction and primer pair 10: 5’-GATCAAGCTTCCTCAGCCTACTATAATGCC-3’ and 11: 5’-CTAGGGATCCCGACAGATGCACTATTAGGC-3’ were used for the second round of reaction [15]. The amplicons were resolved on 1.5% agarose gel. The N-PCR products were gel purified using QIAquick Gel Extraction Kit (QIAGEN GmbH, Hilden, Germany) according to the manufacturer’s instructions. The purified amplicons were subjected to bidirectional sequencing with both forward and reverse primers using BigDye Terminator Cycle sequencing ready reaction kit (Applied Biosystems, Foster City, CA, USA) in ABI 3730 DNA Analyzer (Applied Biosystems). Sequencing was performed at Department of Genetics and Molecular Medicine, Kamineni Life Sciences, Hyderabad.

#### Sequence analysis

The obtained chromatograms were visualized using DNA sequencing software Chromas version 2.6.6 (Technelysium Pty Ltd, Brisbane, QLD, Australia). The forward and reverse primer sequences were assembled using BioEdit version 7.2. The sequence data was identified and compared with preexisting sequence data (as on 29^th^ October 2022) from GenBank, using NCBI BLAST (https://blast.ncbi.nlm.nih.gov/Blast.cgi). All the 49 sequences obtained during this study were deposited in GenBank with accession numbers **OP617281-OP617329**

The reference sequences of *Orientia tsutsugamushi* 56-kDa TSA genes were obtained from GenBank. The reference sequences along with the sequences from this study were aligned using Clustal W (http://www.clustal.org/). Phylogenetic tree with 1000 bootstrap replicates were obtained by Neighbor-joining method and pairwise distances were estimated using the maximum likelihood (ML) method. Phylogenetic tree and evolutionary analyses were conducted in Mega X [16].

### Classification of patients based on severity of illness

The patient data was classified into two groups namely severe and non-severe based on organ involvement and laboratory parameters (Table 1). Patient with any one of the criteria as mentioned in the table was considered as severe.

**Table 1:** Criteria for severe scrub typhus [17].

Organ Involvement	Description of Organ Involvement
Cardiovascular system	Presence of any of the following: a) Systolic blood pressure <90 mmHg or ionotropic support for maintaining blood pressure b) Abnormal cardiac arrhythmia with no previous history of atrial fibrillation, supraventricular tachycardia, or frequent premature ventricular tachycardia c) Myocarditis: Disproportional tachycardia with elevated CK-MB/ serum troponin
Respiratory system	Presence of acute respiratory distress syndrome, defined as follows a) Saturation <92% or PaO_2_/ FiO_2_(mmHg) <200 and infiltrates on chest X-ray
Central nervous system	Presence of any of the following: a) GCS <12 without other identified causes b) Seizure without other identified causes c) Meningitis / meningoencephalitis with CSF analysis showing more than 10 cells
Haematology	a. Platelet count ≤ 20,000 cells/mm^3^
Renal system	a. Creatinine ≥ 2 mg/dL or Creatinine change > 0.5 mg/dL/day at baseline compared to previous values within 1 week.
Hepatic system	Presence of hepatitis, as defined by the following: a. Elevated SGOT/SGPT (AST/ALT) with total bilirubin >2mg/dL

CK-MB, Creatine Kinase-Myocardial Band; GCS, Glasgow Coma Scale; CSF, Cerebrospinal fluid; SGOT, Serum glutamic-oxaloacetic transaminase; SGPT, Serum glutamic pyruvic transaminase; AST, Aspartate aminotransferase; ALT, Alanine transaminase.

### Statistical analysis

Statistical analysis was performed using IBM SPSS Version 20.0 for Windows (SPSS IBM, Armonk, NY, USA). Descriptive variables were described in terms of counts, percentages for all categorical variables. Continuous variables which are normally distributed were described in terms of mean ± S.D. Continuous variables which are not normally distributed were described in terms of the median (IQR). Chi-square test was used to compare the distribution of categorical variables across the severe and non-severe scrub typhus groups. Independent sample t-test/Mann-Whitney U test was used to compare the mean/median of continuous variables across severe and non-severe groups. All tests of significance are two-tailed with a p-value less than 0.05 indicating statistical significance. The laboratory parameters (continuous variables) were dichotomized using the laboratory reference range as cut off point, and the unadjusted odds ratio with 95% confidence interval were calculated. The odds ratio was calculated using MedCalc software Version 22.001 (MedCalc Software Ltd, Ostend, Belgium). Since platelet count, total bilirubin, direct bilirubin, aspartate aminotransferase and serum creatinine were considered for the definition of severe scrub typhus, significant parameters were limited and multivariate model for scrub typhus could not be performed. Phylogenetic tree with 1000 bootstrap replicates were obtained by Neighbor-joining method and pairwise distances were estimated using the maximum likelihood (ML) method. Phylogenetic tree and evolutionary analyses were conducted in Mega X [16]. After phylogenetic analysis, the patient data were segregated based on the infecting genotype and subgroup analysis of organ involvement and resource utilization parameters were performed.

### Ethics statement

The study was reviewed by Kasturba Medical College and Kasturba Hospital Institutional Ethics Committee and received approval with number IEC:412/2019. Written informed consent was obtained from all the patients.

## Results

From August 2019 through March 2022, a total of 214 adult patient samples positive for scrub typhus IgM either by ELISA or RDT or combination of these were enrolled into the study. The mean age of the cohort was 48.51 ±14.14 years. Male were 124 (57.94%) and there were 90 (42.06%) women. Among the cohort, farmers were 83 (38.78%), housewives were 69 (32.24%) and daily labourers were 29 (13.55%) among others. The study comprised of patients from 9 districts of Karnataka, with most patients coming from Davangere (28.97%) followed by Shivamogga (15%), Udupi (13.55%), Haveri (11.2%), Chikmagalur (10.28%), Chitradurga (10.28%), Uttara Kannada (6.07%), Bellary (3.27%) and Vijayanagara (0.46%) districts. Two patients in the study were from neighbouring states of Karnataka, with 1 (0.46%) patient from the Kannur district, Kerala and 1 (0.46%) patient from Anantapur district, Andhra Pradesh.

Seasonality of scrub typhus was noted from August to March of the three years, with cases peaking during the months of September through January and rapidly declining from February. The presenting manifestations of the cohort were fever (210, 98.13%), chills (78, 36.44%), myalgia (64, 29.9%), headache (61, 28.5%), vomiting (58, 27.1%), abdominal pain (56, 26.1%), cough (51, 23.8%), and breathlessness (43, 20%). Altered sensorium was seen in 17 (7.9%) patients and 5 (2.33%) patients had seizures. Eschar was noted in 31 (14.4%) patients. Among females, eschar was primarily noted on the abdomen (7, 43.75%) followed by chest (4, 25%). However, males had varied distribution of eschar. When stratified based on severity, eschar was noted in 22 (16.7%) severe patients and 9 (11%) non-severe patients. The presence of an eschar was not significantly associated with severity of scrub typhus infection.

**Fig 1:**
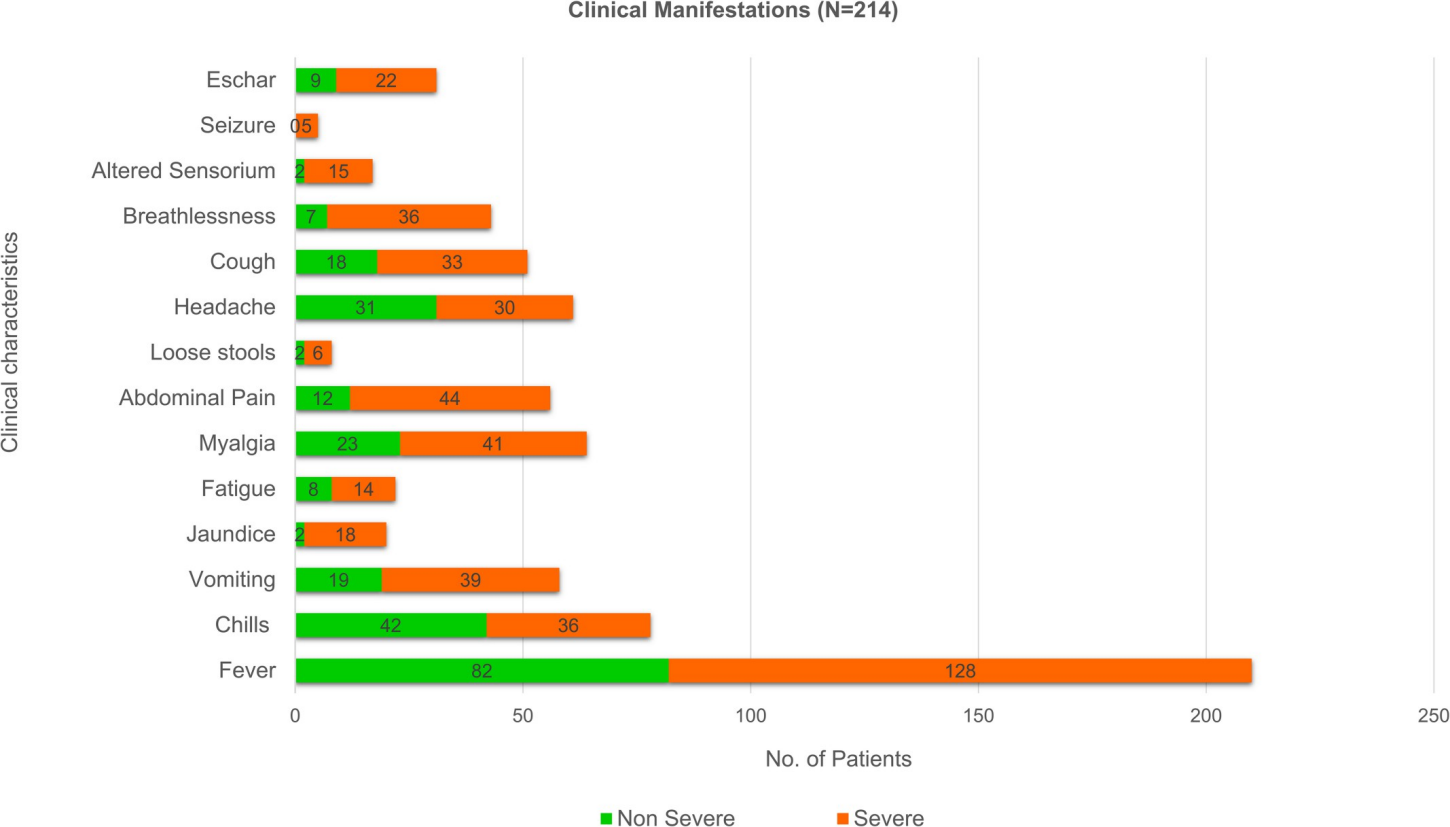
Clinical features of the scrub typhus cohort stratified among severe and non-severe.

The patients enrolled in the study were segregated into severe and non-severe groups based on the criteria mentioned in Table 1. Among the severe group were 132 (61.69%) patients and the non-severe group consisted of 82 (38.31%) patients. Presenting clinical features stratified based on the severity are mentioned in Fig 1.

### Laboratory investigations

There were no significant differences in hemoglobin (Hb) and WBC counts between the groups. However, platelet count of 72,000 (36,500, 1,26,500) among the severe group was significantly deranged. Overall, among the cohort, thrombocytopenia (<150,000 cells/mm^3^) was seen in 99 (46.3%) patients and severe thrombocytopenia (<50,000 cells/mm^3^) was seen in 46 (21.5%) patients. A total of 98 (45.8%) patients had frank hepatitis. Frank hepatitis was defined as AST more than three times the normal laboratory reference range (40 IU/L) which is 120 IU/L.

The laboratory parameters (continuous variables) were dichotomized using the laboratory reference range as cut off point, and the unadjusted odds ratio with 95% confidence interval were calculated. There was a total of eight laboratory parameters, platelet count (<1,50,000 cells/mm^3^), total bilirubin (>1.4 mg/dL), direct bilirubin (>0.3 mg/dL), aspartate aminotransferase (>40 IU/L), alkaline phosphatase (>130 U/L), total protein (<6.4 g/dL), serum albumin (<3.5 g/dL), and serum creatinine (>1.2 mg/dL) that showed a significant correlation with severe scrub typhus (Table 2). Since platelet count, total bilirubin, direct bilirubin, aspartate aminotransferase, and serum creatinine were considered for the definition of severe scrub typhus, significant parameters were limited and a multivariate model for scrub typhus could not be performed.

**Table 2:** The unadjusted odds ratio for laboratory parameters of scrub typhus cohort.

Parameter	Non severe Scrub typhus (N = 82) n (%)	Severe Scrub typhus (N = 132) n (%)	Odds ratio (95% CI)	p-value
Hemoglobin (<12.0 g/dL)	40 (48.7)	67 (50.8)	1 (0.6–1.8)	0.778
Platelet count (< 1,50,000 cells/mm^3^)	39 (47.5)	106 (80.3)	4.5 (2.4–8.6)	< 0.0001
Total White Blood Cells count (> 10,000 cells/mm^3^)	27 (32.9)	47 (35.6)	1.1 (0.6–2)	0.631
Total Bilirubin (>1.4 mg/dL)	11 (13.4)	100 (75.7)	20.1 (9.5–42.6)	< 0.0001
Direct Bilirubin (>0.3 mg/dL)	40 (48.7)	118 (89.3)	8.4 (4.1–17)	< 0.0001
Aspartate aminotransferase (>40 IU/L)	71 (86.6)	125 (94.7)	2.7 (1–7.4)	0.044
Alanine transaminase (>41 IU/L)	69 (84.1)	118 (89.4)	1.5 (0.7–3.5)	0.263
Alkaline phosphatase (>130 U/L)	41 (50)	104 (78.7)	4.1 (2.2–7.6)	< 0.0001
Total Protein (< 6.4 g/dL)	34 (41.4)	92 (69.6)	3 (1.7–5.5)	0.0002
Serum Albumin (<3.5 g/dL)	46 (56)	117 (88.6)	7.1 (3.3–15.4)	< 0.0001
Serum Creatinine (>1.2 mg/dL)	12 (14.6)	54 (40.9)	4 (1.9–8.1)	0.0001

### Organ dysfunction among scrub typhus cohort

**Fig 2:**
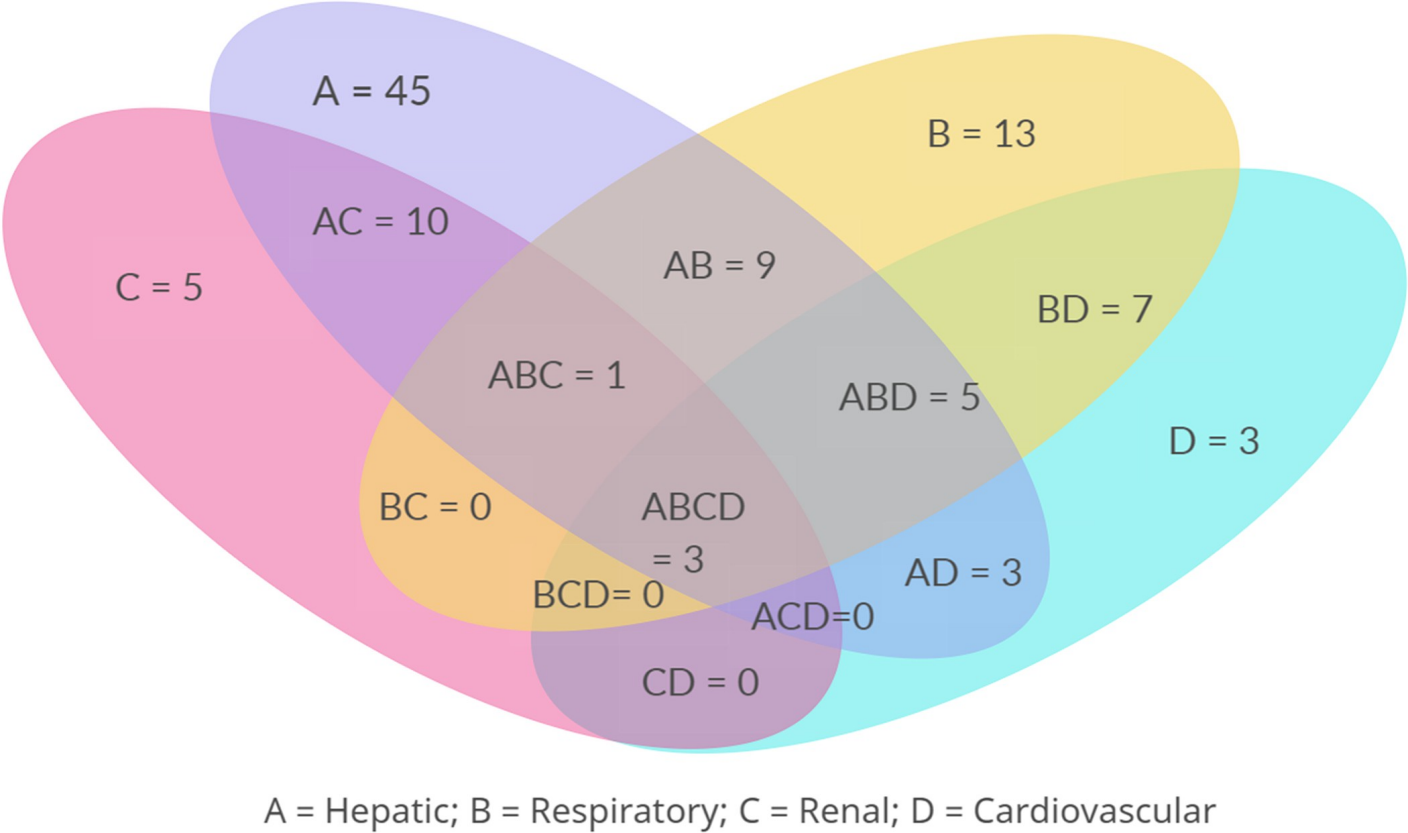
Venn diagram showing overlap and distribution of four major complications in scrub typhus cohort (n = 111).

Among the 132 severe patients (categorised based on Table 1) in the study major organ involvement noted was liver dysfunction in 90 (68.18%) patients. Followed by liver dysfunction was respiratory involvement in 42 (31.81%) patients, renal involvement in 29 (21.96%) patients, cardiovascular involvement in 26 (19.69%) patients, thrombocytopenia in 17 (12.87%) patients and central nervous system involvement in 12 (9.09%) patients. Among 26 patients with cardiovascular involvement, myocarditis and hypotension were seen in 12 (46.15%) patients each and pericardial effusion was seen in 2 (7.7%) patients. Multi organ involvement was seen in 59 (44.69%) patients. Among the patients with multiorgan involvement 77.9% of patients had hepatitis followed by respiratory involvement in 61% of patients. Renal and cardiovascular involvement in patients with multi-organ involvement was 38.98% each. Central nervous system involvement in multi-organ patients was seen in 16.9% of patients. There was one patient with all six-organ involvement, who has survived. While there were two patients with five organs involved, of which one succumbed. Three patients had organ involvement with hepatic, respiratory, renal, and cardiovascular systems (Fig 2).

### *Orientia tsutsugamushi* diagnosis

Eschar was noted in 31 (14.4%) patients. Scrub typhus IgM ELISA was positive in 213 (99.5%) samples. *Orientia tsutsugamushi* real-time PCR was positive in 91 (42.5%) samples. The median duration from onset of illness to sample collection among real-time PCR positive patient samples was 8 (7, 11) days, while for the negative samples was 10 (8, 13) days. N-PCR was positive in 79 (36.9%) samples. The median duration from the onset of illness to sample collection among N-PCR positive patient samples was 8 (6, 11) days, while for the negative samples was 9 (8, 13) days.

### Phylogenetic analysis

**Fig 3:**
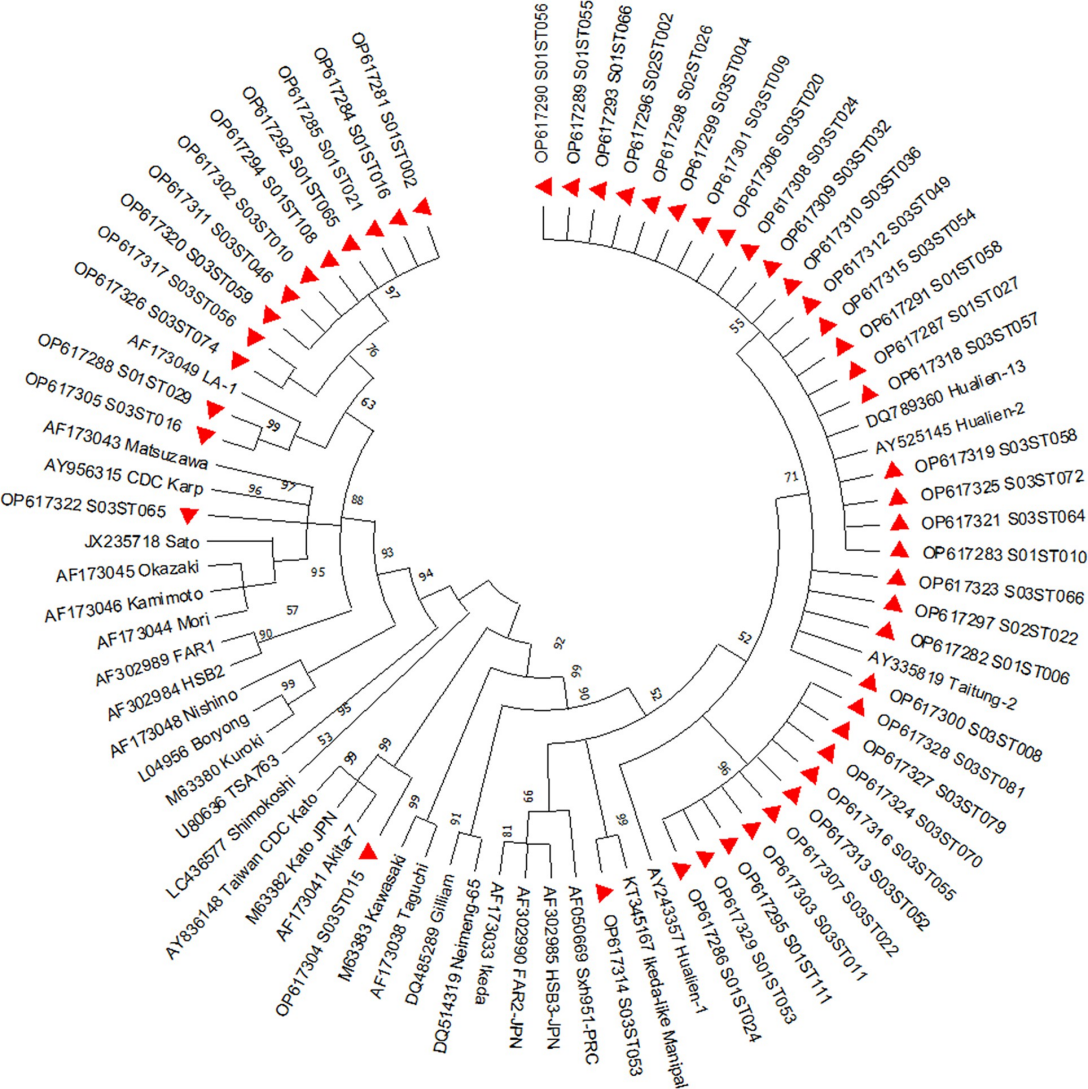
Phylogenetic tree of *Orientia tsutsugamushi* isolates from scrub typhus patients in Karnataka.

A total of 49 sequences with good reads were included in the sequencing analysis for phylogenetic tree preparation. Sequencing analysis revealed most sequences (24, 48.98%) were similar to JG-v (Japanese Gilliam-variant) followed by 13 (26.53%) sequences similar to Karp-related genotype and 10 (20.41%) sequences formed a branch from JG (Japanese Gilliam). One (2.04%) sequence was similar to the Kato genotype, another (2.04%) sequence was similar to Ikeda prototype of the JG cluster (Fig 3).

The evolutionary history was inferred by using the Maximum Likelihood method and Kimura 2-parameter model. Initial tree(s) for the heuristic search were obtained automatically by applying Neighbor-Join and BioNJ algorithms to a matrix of pairwise distances estimated using the Maximum Composite Likelihood (MCL) approach, and then selecting the topology with superior log likelihood value. This analysis involved 79 nucleotide sequences. All positions containing gaps and missing data were eliminated (complete deletion option). Evolutionary analyses were conducted in MEGA X.

Among the sequences similar to JG-v, 20 sequences aligned along with Hualien-2 strain (**AY525145**) and Hualien-13 strain (**DQ789360**), and 4 sequences aligned along with Taitung-2 strain (**AY335819**). Ten sequences from our study formed a subbranch to Hualien-1- ROC (**AY243357**) of the JG cluster. One sequence was similar to the previously described Ikeda-like sequence from Manipal, Karnataka, India. Among the Karp group, one sequence was close to CDC Karp (**AY956315**), while the remaining 12 sequences formed a separate cluster from Karp, of which two sequences formed a cluster with LA-1 (**AF173049**).

Sequencing analysis of the 49 isolates showed nucleotide similarity to be 95.78% to 100% with isolates from five different geographical locations of Vietnam, Bangladesh, and India. Nucleotide similarity of 34 (69.39%) sequences from our study were 99.16% to 100% similar to isolates from Vietnam, 10 (20.41%) sequences of this study were 95.78% to 100% similar to isolates from Bangladesh. The remaining 5 sequences were similar to isolates from India, with 3 (6.12%) sequences being 100% similar to isolates from Manipal, Karnataka, India. One sequence each was 99.53% and 99.38% similar to isolates from Assam, India and Tirupathi, Andhra Pradesh, India respectively.

### Distribution of *Orientia tsutsugamushi* genotypes in districts of Karnataka

The study consists of most sequences from Davangere (17, 34.7%) followed by Chitradurga (8, 16.33%), and with the least number of sequences obtained from Anantapur (1, 2.04%) and Vijayanagara (1, 2.04%) districts. JG-v-like strains were identified in 7 districts of Karnataka during the study period. Followed by JG-v is Karp, noted in five districts and JG was noted in four districts. The only Kato isolate in this study was from the Udupi district (Table 3).

**Table 3:** District wise distribution of *Orientia tsutsugamushi* genotypes.

District	JG-v, n = 24	JG, n = 11	Karp, n = 13	Kato, n = 1	Total, n = 49
Davangere no. (%).	7 (41.17)	4 (23.53)	6 (35.3)	0 (0)	17
Chitradurga no. (%).	5 (62.5)	3 (37.5)	0 (0)	0 (0)	8
Udupi no. (%).	3 (42.9)	0 (0)	3 (42.9)	1 (14.2)	7
Shivamogga no. (%).	3 (60)	0 (0)	2 (40)	0 (0)	5
Chikmagalur no. (%).	0 (0)	3 (75)	1 (25)	0 (0)	4
Bellary no. (%).	2 (66.6)	1 (33.3)	0 (0)	0 (0)	3
Haveri no. (%).	2 (66.6)	0 (0)	1 (33.3)	0 (0)	3
Vijayanagara^a^ no. (%).	1 (100)	0 (0)	0 (0)	0 (0)	1
Anantapur^b^ no. (%).	1 (100)	0 (0)	0 (0)	0 (0)	1

^a^Vijayanagara district was formed as a new district of Karnataka on 2^nd^ October 2021. Only the samples collected after the formation of new district were classified under Vijayanagara district, while all the samples collected prior to formation of new district were classified according to previous districts list of Karnataka, India

^b^Anantapur district is part of Andhra Pradesh, which is a neighboring district of Bellary and Chitradurga districts of Karnataka

### Association between infecting genotype and organ involvement

In our study patients infected with the Karp-like strains had respiratory and cardiovascular involvement followed by severe thrombocytopenia. Patients infected with JG-v-like strains had hepatic, renal and central nervous system (CNS) involvement. Among 132 severe patients, intensive care unit (ICU) admission, ventilation, inotropic support, dialysis, and median length of hospitalisation, 9 (7, 11) days were higher with statistical significance compared to non-severe group. Compared to other genotypes in our study, patients infected with the Karp-like genotype required more resource utilization (Table 4).

**Table 4:** Organ involvement and resource utilization among scrub typhus cohort stratified by infecting genotype.

Variables	JG-v (n = 24) n (%) or median (IQR)	Karp (n = 13) n (%) or median (IQR)	JG (n = 11) n (%) or median (IQR)	Kato (n = 1) n (%) or median (IQR)
Cardiovascular (n = 12)	5 (20.8)	6 (46.2)	1 (9.1)	0 (0)
Respiratory (n = 21)	8 (33.3)	9 (69.2)	3 (27.3)	1 (100)
CNS (n = 4)	3 (12.5)	1 (7.7)	0 (0)	0 (0)
Haematology (n = 9)	5 (20.8)	3 (23.1)	1 (9.1)	0 (0)
Renal (n = 12)	8 (33.3)	4 (30.8)	0 (0)	0 (0)
Hepatic (n = 30)	19 (79.2)	6 (46.2)	4 (36.4)	1 (100)
**Resource Utilization**				
ICU Admission	14 (58.3)	9 (69.2)	3 (27.3)	1 (100)
Ventilation	8 (33.3)	9 (69.2)	2 (18.2)	1 (100)
Ionotropic Support	5 (20.8)	7 (53.8)	1 (9.1)	0 (0)
Dialysis	3 (12.5)	3 (23.1)	0 (0)	0 (0)
Blood Transfusion	6 (25)	4 (30.8)	1 (9.1)	0 (0)
Duration of Hospital Stay	9 (5.75, 10)	9 (4, 12)	8 (5, 10)	9

CNS, central nervous system; ICU, intensive care unit.

### Co-positives and superinfections

If a patient had more than one infection at the time of presentation, it was considered a co-positive. If a patient had acquired a new infection after 48 hours of hospital stay, it was considered as superinfection. Among 91 real-time PCR-confirmed cases of scrub typhus, 22 (24.1%) patients either had co-positive or superinfections or both. Co-positive with Leptospira was seen in 12 (13.1%) patients, followed by Hepatitis B in 2 (2.2%). Superinfection with *Acinetobacter baumannii* was noted in 2 (2.2%) patients, one (1.1%) patient had multidrug-resistant *Escherichia Coli* sepsis and one patient (1.1%) had both *Acinetobacter baumannii* and Leptospira along with scrub typhus (Table 5). All these superinfections were healthcare-associated infections (HAIs). Among the patients with co-positives, 15 (88.2%) patients were classified as severe according to the severity criteria of which, 14 (93.3%) patients required intensive care admission. All the 5 (100%) patients with superinfection required intensive care admission.

**Table 5:** Co-positivity and superinfections among RT-PCR confirmed cases of scrub typhus.

Co-positivity / Super infection	(N = 91) n%
*Acinetobacter baumannii*	2 (2.2)
*Candida tropicalis*	1 (1.1)
Dengue NS1 Ag	1 (1.1)
Dengue IgM	1 (1.1)
Hepatitis B DNA PCR	2 (2.2)
Leptospira IgM	12 (13.1)
Leptospira IgM + *Acinetobacter baumannii*	1 (1.1)
Leptospira IgM + H1N1 RT-PCR	1 (1.1)
MDR *Escherichia Coli* Sepsis	1 (1.1)
**Total**	**22 (24.1)**

NS1 Ag, non-structural 1 antigen; IgM, Immunoglobulin M; DNA, deoxyribonucleic acid; PCR, polymerase chain reaction; MDR, multidrug-resistant.

### Treatment

Among the severe patients 72 (54.5%) received doxycycline, 36 (27.3%) patients received combination of azithromycin and doxycycline, 22 (16.7%) patients received azithromycin, 1 (0.8%) patient received ceftriaxone and 1 (0.8%) patient succumbed to scrub typhus prior to initiation of antibiotics. Among the non-severe patients 54 (65.9%) received doxycycline, 17 (20.7%) patients received combination of azithromycin and doxycycline, 9 (11%) patients received azithromycin and 2 (2.4%) patients received ceftriaxone.

### Outcomes

All the 82 non-severe patients in the study had recovered. Among the 132 severe patients, 122 (92.4%) patients recovered, 4 (3%) patients succumbed in hospital and 6 (4.5%) patients were discharged against medical advice (DAMA) in critical condition. On telephonic follow up, 4 DAMA patients recovered while 2 DAMA patients succumbed. Overall, 6 (2.8%) patients succumbed with 3 (50%) patient samples being similar to Karp-like and the other 3 (50%) were similar to JG-v. Among the 6 patients succumbed, 3 patients had mono-infection with scrub typhus. While one patient was copositive for Leptospira and two patients had MDR *E*.*Coli* and *Candida tropicalis* sepsis. The same patient with *Candida tropicalis* was diagnosed with ventilator-associated pneumonia. These HAIs are preventable conditions contributing to poor outcome.

## Discussion

Scrub typhus is an acute febrile illness, which in a proportion of patients causes multi-organ dysfunction. Multiple studies across different parts of India have reported cases of scrub typhus with a wide variation in circulating genotypes. Gilliam-like strains were reported from Bihar [12]. Shimla from the Northern part of India reported an equal proportion of Karp and Kato-like strains. While Shillong which is part of the Northeast and Vellore, Tamil Nadu, which is located close to our study setting had a preponderance of Kato-like strains [7]. In contrary, our study had only one (2.04%) Kato-like strain which formed a separate branch within the Kato cluster. A predominance of Karp-like strains (64.7%) was seen in Haryana, Punjab, Uttar Pradesh, Himachal Pradesh, and Chandigarh of North India [18]. However, in our study only 26.53% of sequences were Karp-like. Similarly, Karp-like sequences were reported from Vellore (18.9%) and Shillong (23.3%) in a smaller proportion. Ikeda, which was initially reported from Japan, was seen among isolates from Vellore and Shimla [7]. Koraluru et al., also have identified Ikeda-like strain in Manipal [11]. During phylogenetic analysis, one of our isolates has formed a separate branch along with Ikeda like strain (**KT345167**) reported by Koraluru et al., far from the original Ikeda prototype strain (**AF173033**). In Bangalore, the predominance of strains similar to the Gilliam prototype were seen followed by Kato and Boryong-like isolates [13]. Though our study setting is close to Bangalore, none of the isolates in our study were similar to Boryong-like strains. However, our study consisted of most sequences of JG-v (48.98%) and JG (22.45%) of Japanese Gilliam cluster. Previously JG like strains were reported from Himachal Pradesh and Tirupathi, Andhra Pradesh in India and globally reported from Japan, China, and Southeast Asia [10, 19, 20]. Neimeng-65 isolates reported from Shillong [7], were not seen in our study.

Pathogen factors and strain variations leading to varying clinical presentation, organ involvement and outcome have been discussed in various in vivo and clinical studies [21–24]. In BALB/c mice, Karp was found to be lethal, while mice induced with Kuroki strains survived [24]. In another study performed in BALB/c mice, the fatality rate varied between two different Boryong strains, suggesting fatality rates might be dependent on both serotypes and virulence genes [23]. In the human population, there are a few studies that have reported differences in clinical features across genotypes. In the Korean population, differences in clinical characteristics, laboratory parameters, eschar (97% vs 73.7%) and skin rash (94% vs 68.4%) were noted among Boryong and Karp-infected patients. General weakness and conjunctival injection, erythrocyte sedimentation rate (ESR) and plasma fibrinogen were comparatively higher among Boryong infected group of patients [22]. In patients from Puducherry and Tamil Nadu, India, isolates close to Vellore were causing less severe infection with a narrow spectrum of infection and isolates close to Taiwan were causing much severe infection with multi-organ involvement including ARDS and hypotension. Karp was associated with hepatitis, meningitis and multiorgan dysfunction syndrome [21]. In contrast, Karp infected patients in our study had respiratory and cardiovascular involvement followed by severe thrombocytopenia. Patients infected with JG-v had hepatic, renal and CNS involvement in our study. Host and pathogen factors both have a role in organ involvement and severity of infection. Host factors such as humoral and cellular immune responses might play a role in vasculitis, leading to organ involvement and severity of infection. Pathogen factors such as different strains of *Orientia tsutsugamushi* may lead to varying disease severity [25]. In BALB/c mice, virulence of Karp and Kuroki strains depended on their ability to escape phagosomes and survive killing in the host cytoplasm [24]. Further studies on host and pathogen factors might give a better understanding of the factors associated with varied organ involvement and infecting strain.

Eschar was noted in 31 (14.4%) patients in our cohort. The incidence of eschar varied across different studies [2, 5, 22, 26]. The occurrence of eschar may depend on the infecting genotype [22]. The primary site of eschar among females was the abdomen (7, 43.75%) followed by the chest (4, 25%). This finding is similar to a previous report [26]. Eschar sites among males differed in our study. Compared to patients from Vellore [5], multi organ involvement was slightly higher in our study (34% vs 44.69%). Varghese et al., have reported, among patients with MODS, hepatitis (total bilirubin >2mg/dL) had a lower prevalence of 63.7% vs 77.9% and respiratory involvement had a higher prevalence of 76.9% vs 61% which is in contrast to our findings [5]. Thrombocytopenia (<150,000 cells/mm^3^) in the Indian population was noted to be in a wide range of 10% - 70.7%, while severe thrombocytopenia (<50,000 cells/mm^3^) were noted in 27%– 46.2% of patients [1, 27, 28]. Similarly in our cohort thrombocytopenia was seen in 46.3% patients. However severe thrombocytopenia was seen in a slightly lower number of patients (21.5%). Mortality of scrub typhus in India is 6.3% [29], while we had observed a much lower mortality of 2.8%. The improvement in mortality trend could be due to early recognition of scrub typhus cases and timely initiation of antibiotics. In scrub typhus endemic regions, serological co-positives have been reported [12, 30]. However, these serological co-positives could be cross-reactivity, which require confirmation with molecular tests or appropriate gold standard tests.

Our study has certain limitations. Among the cohort 11 (5%) patients were diagnosed and administered with antibiotics prior to referral to our centre. The median duration from onset of illness to sample collection of 214 patients in the study was 9 (7, 12) days. The median duration from onset of illness to sample collection among real-time PCR negative samples was 10 (8, 13) days. Prior intake of antibiotics and delay in presentation to our hospital might have led to low positivity in real-time PCR (42.5%) and N-PCR (36.9%) in our study. Not all N-PCR positive samples were docile for good sequencing. Due to this, we might have underrepresented the circulating genotypes or there is a small chance that we could have missed other genotypes present in this region. Our study consisted of only 49 sequences, which comprised of majorly 3 genotype groups (JG-v, Karp, and JG) while only one sequence was similar to Kato genotype. To establish an association between infecting genotype and organ involvement, a larger cohort representing all major circulating genotypes is required. Proportion of severity noted in our study is high. This may be because our hospital is a tertiary referral centre, which might receive sicker patients. Further, the severity criteria adopted in our study is from Intravenous Treatment for Scrub Typhus (INTREST) clinical trial, which has bilirubin >2 mg/dL as one of the severity criterion [17]. This might have overestimated the severity even though some of such patients did not require ICU care. Future studies must develop robust severity criterion for scrub typhus not just based on biochemical parameters but also considering patients need for intensive care. Further nation-wide molecular characterization studies need to be carried out considering all the scrub typhus endemic regions. The better the genetic diversity of *Orientia tsutsugamushi* is understood, the better could be the inclusion of proteins or antigens of circulating genotypes for development of region-specific diagnostic kits and development of vaccine candidates.

## Conclusion

In the current study, multi-organ involvement was seen in 44.69% of scrub typhus patients. Hepatitis (77.9%) followed by respiratory involvement (61%) were common among patients with multi organ involvement. JG-v like strains predominated in Karnataka followed by Karp-like strains. Karp-like strains caused much severe form of infection and patients infected with Karp-like strains utilized more hospital resources.
